# *Clostridium perfringens* Epsilon Toxin Targets Granule Cells in the Mouse Cerebellum and Stimulates Glutamate Release

**DOI:** 10.1371/journal.pone.0013046

**Published:** 2010-09-30

**Authors:** Etienne Lonchamp, Jean-Luc Dupont, Laetitia Wioland, Raphaël Courjaret, Corinne Mbebi-Liegeois, Emmanuel Jover, Frédéric Doussau, Michel R. Popoff, Jean-Louis Bossu, Jean de Barry, Bernard Poulain

**Affiliations:** 1 Centre National de la Recherche Scientifique, Institut des Neurosciences Cellulaires et Intégratives UPR3212, Strasbourg, France; 2 Unité des Anaérobies et Leurs Toxines, Institut Pasteur à Paris, Paris, France; The University of Queensland, Australia

## Abstract

Epsilon toxin (ET) produced by *C. perfringens* types B and D is a highly potent pore-forming toxin. ET-intoxicated animals express severe neurological disorders that are thought to result from the formation of vasogenic brain edemas and indirect neuronal excitotoxicity. The cerebellum is a predilection site for ET damage. ET has been proposed to bind to glial cells such as astrocytes and oligodendrocytes. However, the possibility that ET binds and attacks the neurons remains an open question. Using specific anti-ET mouse polyclonal antibodies and mouse brain slices preincubated with ET, we found that several brain structures were labeled, the cerebellum being a prominent one. In cerebellar slices, we analyzed the co-staining of ET with specific cell markers, and found that ET binds to the cell body of granule cells, oligodendrocytes, but not astrocytes or nerve endings. Identification of granule cells as neuronal ET targets was confirmed by the observation that ET induced intracellular Ca^2+^ rises and glutamate release in primary cultures of granule cells. In cultured cerebellar slices, whole cell patch-clamp recordings of synaptic currents in Purkinje cells revealed that ET greatly stimulates both spontaneous excitatory and inhibitory activities. However, pharmacological dissection of these effects indicated that they were only a result of an increased granule cell firing activity and did not involve a direct action of the toxin on glutamatergic nerve terminals or inhibitory interneurons. Patch-clamp recordings of granule cell somata showed that ET causes a decrease in neuronal membrane resistance associated with pore-opening and depolarization of the neuronal membrane, which subsequently lead to the firing of the neuronal network and stimulation of glutamate release. This work demonstrates that a subset of neurons can be directly targeted by ET, suggesting that part of ET-induced neuronal damage observed in neuronal tissue is due to a direct effect of ET on neurons.

## Introduction

Epsilon toxin (ET) is a protein of 30 kDa produced by *Clostridium perfringens* types B and D with a very high lethality (∼400.000 mouse LD_100_/mg protein). This ranks this toxin among the 10 most potent poisonous substances so far known. Infection with the bacteria occurs via food, water, animal litter or soil, and causes severe, often fatal, enterotoxaemia (*e.g.* pulpy kidney disease and diarrhoea) in sheep, goats, cattle, poultry and pigs [1]. ET is secreted in the gut lumen as a proto-toxin and following its activation by endoproteases the toxin compromises the intestinal barrier [2]. This allows ET to spread through the blood-stream, affecting the lungs, kidneys and the brain [1], [3], [4].

ET shares significant sequence homology and structural similarities with aerolysin from *Aeromonas hydrophila* and with alpha toxin produced by *C. septicum* and belongs to a large family of pore forming bacterial toxins [5]–[7]. Most of its cellular mode of action has been deduced from studies performed on renal cell lines or purified membranes. After binding to specific, yet unknown, membrane acceptor(s) ET undergoes a cholesterol-dependent heptamerization leading to the formation of a transmembrane pore [8]–[12]. The channel-forming domain of ET has been recently identified [13]. The ET-induced pore leads to an efflux of K^+^, and influx of Na^+^ and Cl^−^ [8], [11], [14], [15]. In renal cells ET also induces intracellular Ca^2+^ rise, ATP depletion and cell death, which involves a caspase-independent process [12]. However, the causal link between pore formation and altered functions remains unclear: in conditions that prevent ET heptamerization ET can cause cell death [12].

ET-intoxicated animals express severe neurological disorders [1], [4], [16], [17] associated with a marked increase in neurotransmitter release (including glutamate and dopamine) and neuronal cell death [18]–[21]. Altered neurons are found scattered among apparently normal nerve cells in the cerebral cortex, hippocampus, thalamus, basal ganglia and cerebellum; cerebellum is a predilection site for the induction of early central nervous system damage [4], [18], [22].

Since ET binds to capillary endothelial cells and alters the blood brain barrier [23]–[25], the nerve tissue damages caused by ET have been proposed to indirectly result from vasogenic edema [4]. The possibility that ET acts directly on the nerve tissue cells needs to be considered. Several studies support such a possibility: the bilateral symmetry of the damage caused by ET, notably in the brain stem [22], and the local nerve-tissue alterations produced by intra-hippocampal injection of ET [19] suggests a nerve-tissue vulnerability to ET. However, the identity of the cells directly altered by ET remains a matter of debate. On one hand, ^125^I-labelled ET binds and forms pores in membranes purified from rat brain synaptosomes [11], [14], [26]. On the other hand, the most recent studies performed using GFP-tagged ET support the notion that the toxin binds to cells belonging to the glial lineage (the astrocytes and myelin, which is formed by oligodendrocytes), but not to the neurons [25], [27].

In this study, we addressed the possibility that ET targets neurons. To identify the nerve tissue regions of interest, we examined ET-binding on brain slices by immunofluorescence. An intense staining was observed in the cerebellum. By analyzing the staining of ET and cell markers of interest we established that ET binds to the cell body and dendrites of granule neurons and oligodendrocytes, but not to the astroglial cells, GABAergic neurons, or nerve terminals in the cerebellar cortex. To investigate the effect of ET on neurons, electrophysiological and pharmacological analyses were performed and revealed that ET directly depolarizes granule cell somata, thereby greatly stimulating their firing activity and ensuing glutamate release. However, we found that ET has no direct effect on inhibitory cerebellar GABAergic interneurons and Purkinje cells. The effect of ET on glutamatergic neurons is likely to enhance the highly potent action of the toxin, and this may explain why ET lethality is 100-fold higher than that of other structurally related, pore-forming toxins.

## Results

### ET binds to defined regions of the cerebellar cortex

**Figure 1:**
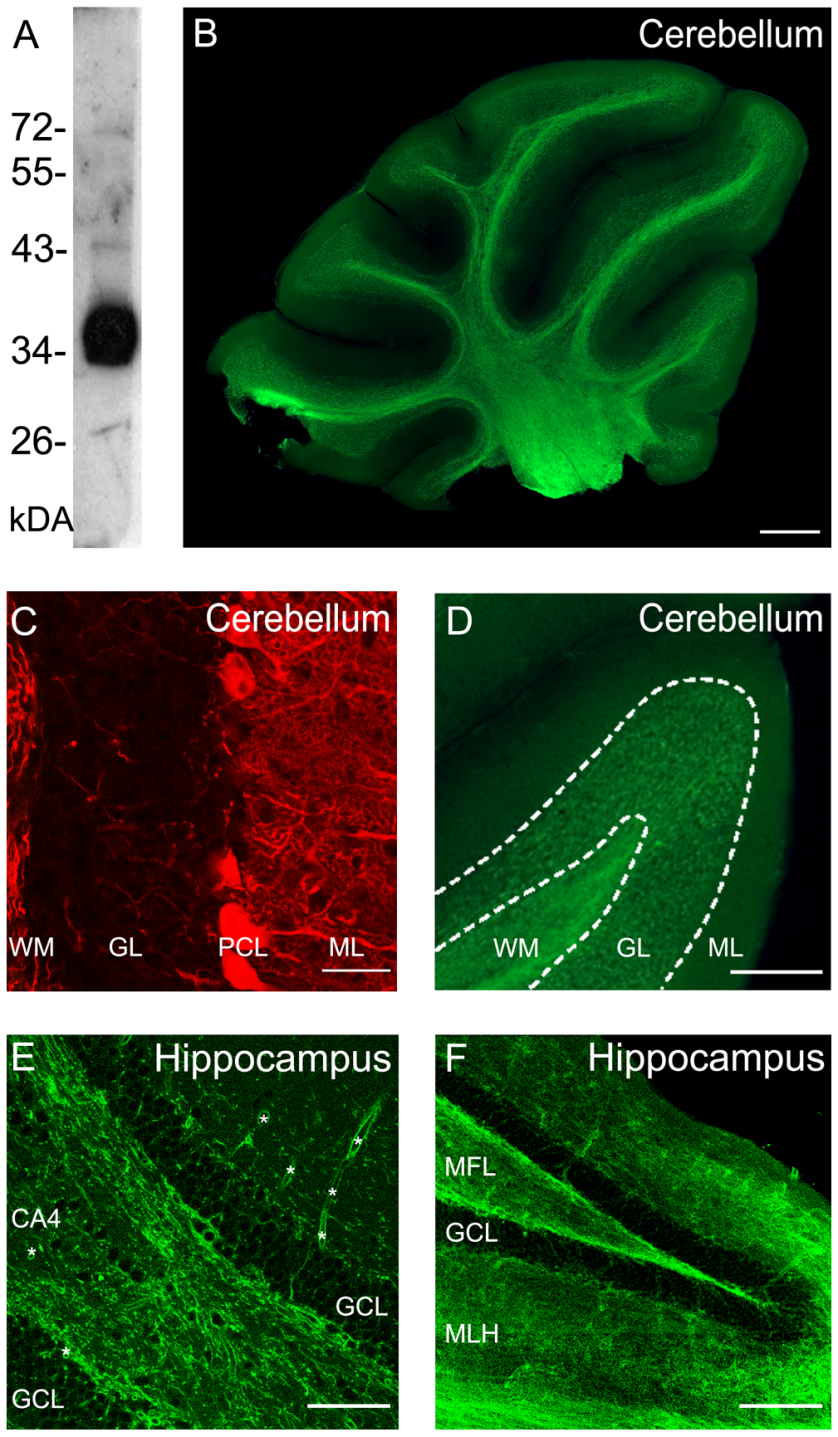
ET staining in mouse cerebellum and hippocampus slices. A, Western blot of rabbit anti-epsilon toxin antibodies. Crude culture supernatant of *C. perfringens* type D was submitted to immunoblotting experiment using immunoaffinity purified rabbit anti-epsilon toxin (1∶2000). Molecular weight markers are indicated. The labeled band at 36 kDa corresponds to ET. ET-immuno staining obtained when tissue slices were incubated with (B, D–F) or without (C) ET (10^−7^ M) for 5 min before fixation. B, mouse cerebellum. Scale bar  = 500 µm. C, Immuno-staining obtained in absence of ET: cerebellar cortex was submitted to double immunostaining against ET and calbindin, which is expressed by the Purkinje cells in their dendrites (i.e. in the molecular layer), cell body, and axons (present in granule cells layer and white matter). Scale bar  = 40 µm. D, Magnification of a cerebellar lobule from (B); Scale bar  = 250 µm. C–D: WM: white matter, GL: granule cells layer, PCL: Purkinje cells layer, ML: molecular layer. E and F, ET-staining obtained in hippocampus: in the CA4 region (E) and in dentate gyrus (F); Scale bars are 50 and 100 µm, respectively. E–F: CA4 denotes presence of large pyramidal cells, MFL: mossy fiber layer, GCL: granule cell layer, MLH: molecular layer of hippocampus, *: capillary blood vessel.

Whole brain acute sagittal slices taken from adult (P25–P30) mice were incubated in the presence of ET (10^−8^ and 10^−7^ M, at room temperature) for 5 min before fixation and then immuno-stained for ET using immunoaffinity purified primary antibodies specific for ET (see single 36 kDa ET band in Fig. 1A). In the presence of 10^−7^ M ET the staining was marked in several brain regions, including cerebellum (Fig. 1B), hippocampus, thalamus, striatum (myelinated tracts), olfactive bulb, the colliculi, and cerebral white matter. To determine possible cross reactivity of the anti-ET antibodies, similar experiments were repeated with slices incubated without ET: no immuno-staining was detected for ET, while immunoreactivity for cells markers could be revealed (Fig. 1C). Figure 1B and D, illustrates ET staining observed in cerebellum at different magnifications, and figure 1D–E shows examples of ET labeling in hippocampus. At 10^−8^ M, the labeling was faint. Overall the staining pattern in the brain is reminiscent of the distribution of tissue injuries (hippocampus, basal ganglia, internal capsule, thalamus and substantia nigra, and cerebellum) resulting from subacute intoxication of mice and rats [4], [18]. The marked staining observed in the cerebellum is consistent with the previous reports that the cerebellum is a predilection site for the induction of ET damage [4], [18], [22].

When ET-labeling was observed at a higher magnification, we found that the toxin markedly stained the capillary vessels (denoted by * in Fig. 1E), numerous fibers likely to correspond to myelinated axons (as in the cerebellar white mater in Fig. 1D or hippocampal regions displayed in Fig. 1E and F). A less intense ET labeling was also found on pericaryon of numerous cells (for example in the granule cell layer in the hippocampus (Fig. 1E) raising the possibility that neurons might be labeled by ET.

To determine whether neurons or a subset of neurons are targeted by ET in the central nervous system we focused on the cerebellar cortex which segregates glial cells and neuron somata or/and processes in identified layers by its laminar organization. When ET was applied (10^−7^ M, at room temperature) for 5 min either before (Fig. 1B, see D for a closer view) or after fixation (not illustrated), the staining distribution was qualitatively similar, albeit less intense when performed on nerve tissue fixed prior to ET application. With the exception of some capillary walls (not illustrated), no obvious structure in the molecular layer was significantly stained by ET (Fig. 1B,D). This indicates that the cells constituting this layer (i.e. the inhibitory GABAergic neurons and the astrocyte related Bergmann's glial cells), as well as neuronal processes, corresponding to parallel fibers from granule cells and dendritic trees of Purkinje cells, are not targeted by ET. The large soma of the Purkinje cells that lie at the interface of the molecular and granule cell layers were not stained (Fig. 1B,D). The granular layer was clearly stained, thus raising the question of which cell types and/or processes are targeted by ET: granule cells, GABAergic interneurons, the nerve fibers afferent to the granule cells (i.e. the mossy fiber nerve terminals), astrocytes or oligodendrocytes. The cerebellar white matter mainly composed of myelinated axons (including the Purkinje cells axons) and oligodendrocytes displayed a strong ET staining.

### Granule cells and oligodendrocytes are the cell targets of ET in the cerebellum

To identify the cells types targeted by ET in the granular layer, we analyzed cerebellar slices immuno-labeled for ET and well defined cell markers.

Since, as mentioned above, the ET-labeling intensity produced by applying the toxin on acute (i.e. still living) slices was higher than that observed when applying ET on fixed tissue, we analyzed double (ET, cell marker) staining in slices submitted to ET 10^−7^ M for 5 min before washing out and fixation (see the Material and Methods). However such a staining procedure can introduce a bias if ET is cytotoxic for a subset of its targets, which is revealed by the fast appearance of pyknotic nuclei in renal cell culture [12] and nerve tissue cells [22]. Accordingly we examined nuclei in the granular layer using the DNA marker DRAQ5. We did not observe any pyknotic nucleus indicating that ET (10^−7^ M for up to 20 min before fixation) does not induce cell death in the acute cerebellar slices at least within the duration of the experiments. This ruled out the possibility that cell-specific cytotoxicity hampers the interpretation of double stained cells.

**Figure 2:**
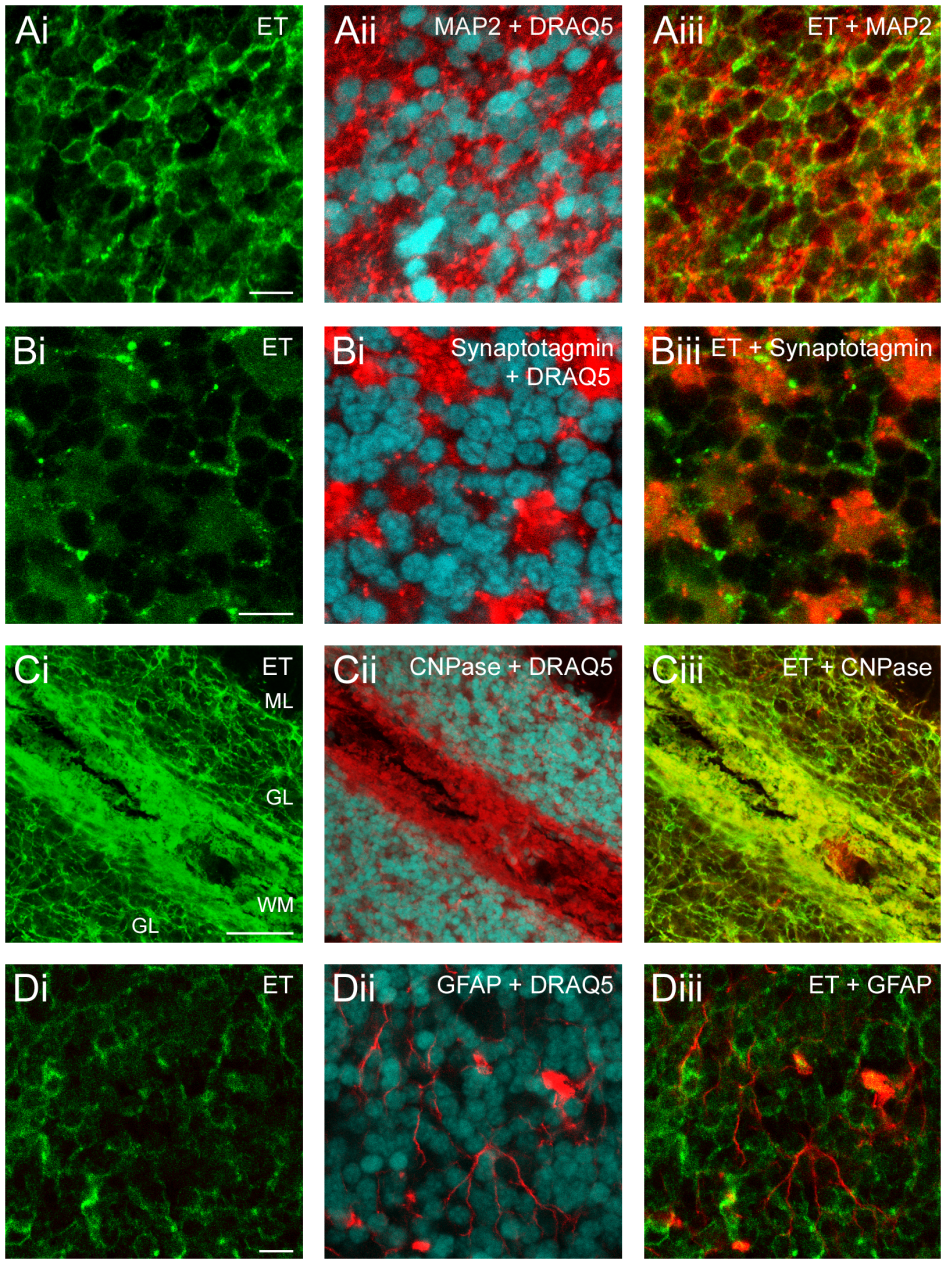
ET stains granule cells and oligodendrocytes but not astrocytes or nerve endings. A–D: column i: ET-staining (green), column ii: specific cell-marker immunoreactivity (red) and DRAQ5 DNA signal (cyan), column iii: merge of the ET and cell-marker immunoreactivities. In all experiments ET was applied for 5 min 10^−7^ M. A: ET and MAP-2, B: ET and synaptotagmin. C: ET and CNPase. D: ET and GFAP. Scale bars are 10 µm in A, B, D, and 50 µm in C.

Each of the double staining experiments (ET, cell marker of interest) listed in Table 1 was performed a minimum of 3 times. Figure 2 illustrates several typical examples. The biological significance of the double staining for each of the mentioned pairs (ET, cell marker) was determined by calculating the Pearson's correlation coefficient (*r_p_*) for each analyzed region of interest (ROI) in multiple slices. The number of analyzed ROI, averaged *r_p_*, and statistical significance are reported in Table 1.

**Table 1:** The double staining by ET and specific cell markers.

	Neurons				Synapses and dendrites				Glial cells		
	Granule cells		Purkinje cells	PC + Interneur.	Pre-synaptic		Post-synaptic	Dendrites	Astrocytes	Oligodendrocytes	
Marker	Alpha-6	Kv3-1b	calbindin	Parvalb	Sytg	Syph	PSD-95	MAP-2	GFAP	CNPase	
ROIs	GL	GL	PCL + ML	PCL + ML + GL	GL	GL	GL	GL	GL	GL	WM
*r_p_* ± SEM	**0.39**±0.03	**0.48**±0.03	**0.002**±0.05	**0.02**±0.04	**−0.05**±0.01	**0.01**±0.01	**0.02**±0.05	**0.22**±0.02	**0.10**±0.02	**0.52**±0.01	**0.36**±0.04
nb. ROI	11	16	8	6	6	6	6	18	6	6	3
**significance**	***	***	***n.s.***	***n.s.***	***n.s.***	***n.s.***	***n.s.***	**/***	***n.s.***	***	**/***
**ET staining**	**yes**	**Yes**	**no**	**no**	**no**	**no**	**no**	**yes**	**no**	**yes**	**yes**

Among the different cerebellar neuron types, granule cells were identified by their expression of the alpha-6-GABA_A_ receptor subunit (alpha-6) [47] or potassium channel subunit Kv3.1b [48]. Purkinje cells were identified by their typical morphology and presence of the Ca^2+^-buffering proteins calbindin and parvalbumin (parvalb), whereas the other GABAergic cerebellar interneurons were identified by expression of parvalbumin but not calbindin [49]. Nerve terminals (*i.e.* pre-synaptic compartment) were identified by the presence of the synaptic vesicle protein synaptotagmin (sytg) [50] or synaptophysin (syph) [51]; whereas Post-Synaptic Density 95 kD protein (PSD-95) was used to label the post-synaptic (i.e. the dendritic spines) compartment [52]. Cerebellar neuronal dendritic trees were identified by the presence of the microtubule associated protein-2 (MAP-2), which is denser in the dendrites [53]. Astrocytes and radial Bergman's glia were identified by Glial Fibrillary Acidic Protein (GFAP) [54], [55]. Oligodendrocytes were identified by expression of 2′,3′-Cyclic Nucleotide 3′-Phosphodiesterase (CNPase) [56]. Analyzed ROI are: ML, molecular layer; PCL, Purkinje cells layer; GL, granular layer; WM, white matter. The averaged Pearson's coefficients (*r_p_* ± SEM) were determined from the mentioned number of ROI. Since the *r_p_* varied from ROI to ROI, we tested the significance of eventual differences in average *r_p_* in all the reported conditions by a pairwise multiple comparison procedure.

ET stained the pericaryon of numerous small MAP-2-positive cells (i.e. neurons) corresponding to granule cells, the most prominent cell type in the granular layer (>3.10^6^ granule cells per mm^3^ in the rat) [28] (Figure 2Ai). Data presented in Table 1 confirms this identification because the highest *r_p_* values (ranging between 0.39 and 0.48) in the granular layer were obtained for the co-staining of ET with two granule cell markers (the alpha-6-GABA_A_ receptor subunit and the potassium channel subunit Kv3.1b). Table 1 shows no significant ET-staining on Purkinje cells, which are calbindin and parvalbumin positive, or GABAergic interneurons, which are calbindin negative but parvalbumin positive, as revealed by almost null *r_p_*. We also determined that ET did not bind to the pre- and post-synaptic compartments in the granular layer as revealed by almost null *r_p_* when ET labeling and synaptotagmin (Fig. 2B), synaptophysin or PSD-95 staining were analyzed (Table 1). Analysis of CNPase and ET co-labeling revealed that ET binds to the oligodendrocytes (Fig. 2C) in agreement with positive and reproducible *r_p_* values (0.52 in the granular layer, and 0.36 in the white matter). Figure 2Ci shows that in the granular layer the CNPase positive structures (i.e. the oligodendrocytes and their processes) were stained with higher intensity than that observed on granule cells.

Whatever the staining conditions (ET applied before or after tissue fixation), the ET and GFAP co-labeling was low (Fig. 2D), with an averaged *r_p_* = 0.10. This value is close to that obtained when analyzing co-staining of pairs of markers specific for distinct cell types: for example, immunolabeling against alpha-6-GABA_A_ (specific of the granule cells) and GFAP (specific of the astrocytes) led to an average *r_p_* = 0.15±0.03, *n* = 6. Thus, the averaged *r_p_* = 0.10 found for the ET GFAP co-staining does not indicate that ET binds to the astroglial cells and likely results from interspersion of astrocytes with other cell types present in the cerebellar tissue giving rise to detection of signals from different cell types in a small portion of the analyzed voxels.

To summarize the above data establishes that ET targets granule cell somata, which are glutamatergic neurons, but not the granule cell axons or nerve terminals, afferent nerve terminals or cerebellar GABAergic neurons (Purkinje cells or interneurons). Among the glial cell lineage, ET binds to oligodendrocytes, but neither the astrocytes nor related radial glia (i.e. Bergman's cells).

### ET effects on granule cells in primary culture

**Figure 3:**
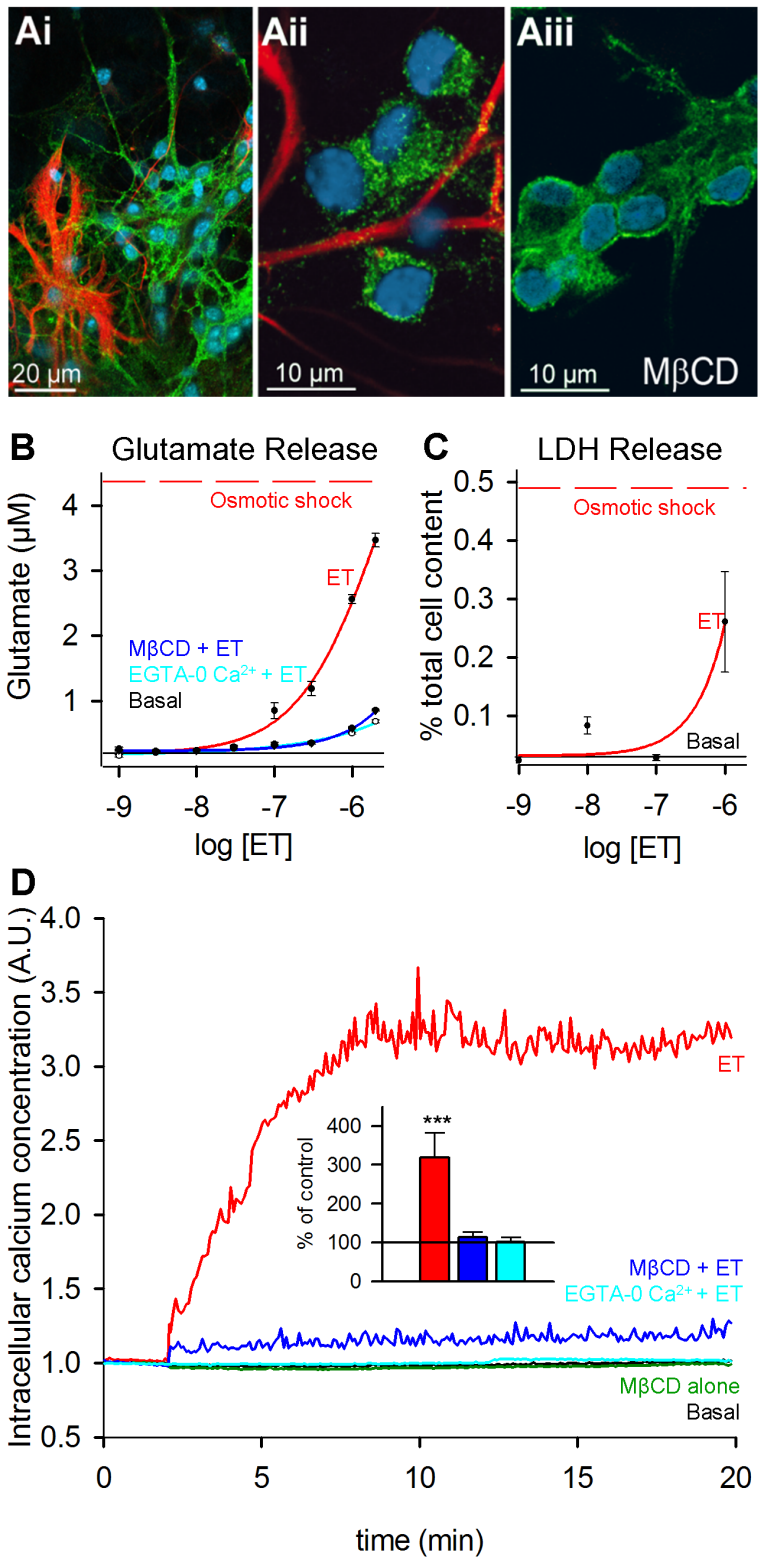
Effect of ET on primary cultures of cerebellum highly enriched in granule cells. Ai, Aii and Aiii, primary cultures of cerebellar cells. Ai and Aii, 10^−7^ M ET was applied in PBS buffer (i.e. containing no Ca^2+^ ions) for 5 min after fixation of the cells, then the ET- (green) and GFAP-(red) immunoreactivities were revealed. Cell nuclei were labeled using DRAQ5 (cyan). GFAP-positive cells (astrocytes) are not labeled by ET. Aiii, same kind of experiment except that the culture was pretreated with methyl-β-cyclodextrin (MβCD, 1 mM for 30 min at 37°C) before fixation. B, the glutamate concentration (µM) in extracellular medium was determined from cerebellar primary cultures using the Amplex Red Assay. ET was applied for 10 min at the indicated final concentrations, under the following conditions: ET alone (red) ET after pretreatment with methyl-β-cyclodextrin (blue), ET in physiological medium containing no Ca^2+^ (10 mM EGTA; 0 mM CaCl_2_; cyan). Data points are mean ± SEM determined from triplicate determinations. C, extracellular lactate-deshydrogenase (LDH) levels (using the Cytotoxicity Detection Kitplus assay). Data points are mean ± SEM (*n* = 4). In B and C, lower black horizontal lines denote basal levels (i.e. without ET), and upper dashed red lines denote the maximal values determined after granule cell lysis was induced by hypo-osmotic shock. D, averaged Fura-2 measurements of intracellular [Ca^2+^] from 25 granule cells, in absence (black, denoted as Basal)) or after addition of 10^−7^ M ET (red), with ET after preincubation of the cells with methyl-β-cyclodextrin (1 mM for 30 min, blue), or with medium containing 10 mM EGTA-0 mM Ca^2+^ (cyan). The green curve shows control cells preincubated only with methyl-β-cyclodextrin. Inner graph: relative [Ca^2+^] changes (% of control) determined 10 min after addition of ET in absence (red, n = 15) or presence of methyl-β-cyclodextrin (blue, n = 5) or in EGTA-0 Ca^2+^ medium (cyan, n = 14). ET *vs* control: *p*<0.001, ET + MβCD *vs* control or MβCD alone: *n.s.*

Having established that ET binds granule cells in cerebellar slices, we performed experiments aimed at examining the specific effect(s) of ET on granule cells in primary culture. Under the conditions used to prepare primary cultures of mice cerebellar cortex, most of the cells present are granule cells (96%), few are astrocytes (<4%) and none are oligodendrocytes, Purkinje cells or fibroblasts. When applied at 10^−7^ M in PBS onto fixed primary cultures, ET stained granule cells, but not astrocytes, which express GFAP (Fig. 3Ai, and 3Aii for a closer view), confirming the observations made above. Since PBS buffer does not contain Ca^2+^ ions, this indicates that binding of ET to granule cells does not require Ca^2+^ ions. When applied at lower concentrations (10^−8^ M), no significant ET labeling was observed. A noticeable difference compared with the staining of ET in acute slices is that the toxin binds to the neuronal somatic and neurite membranes as well as varicosities corresponding to nerve terminals on the cultured neurons (not illustrated).

Since the ET injection into the brain stimulates glutamate and dopamine release, by a yet unknown mechanism [19]–[21], we first examined whether ET could trigger glutamate release from granule cells. Figure 3B shows a typical experiment of a series of 5 during which increasing concentrations of ET were tested. Consistent with the staining of granule cells by ET that we observed at 10^−7^ M, but not 10^−8^ M, ET 10^−7^ M and higher concentrations, but not 10^−8^ M and lower ones, induced glutamate efflux (red, Fig. 3B). This effect was strongly diminished when the experiments were repeated using physiological medium lacking Ca^2+^ ions (containing 10 mM EGTA, and 0 mM CaCl_2_; cyan curve in Fig. 3B). Since ET binds to granule cells in absence of Ca^2+^ ions (see above, and data not shown), this finding suggests that glutamate release is mediated by a Ca^2+^-dependent process. However, we also detected leakage of cytoplasmic lactate deshydrogenase (LDH) in the surrounding medium (Fig. 3C). Consistent with previous reports [19]–[21] these experiments indicate that ET causes glutamate efflux from cultured granule neurons in part due to stimulation of the Ca^2+^-dependent glutamate release machinery and in part due to neuronal leakage as indicated by the release of LDH. This latter effect might account for presence of a Ca^2+^-independent glutamate release component when ET is applied at high concentration. Moreover, we observed that after a 10–20 min incubation with ET 10^−7^ M, many granule cells presented membrane blebs (not illustrated). This manifestation of late ET cytotoxicity on the cultured granule cells contrasts with the apparent lack of detectable cell toxicity in acute slices (see above mention of lack of appearance of pyknotic nuclei).

In the renal cells lines, ET has been reported to induce a rise in the intracellular concentration of [Ca^2+^] [12]. Similarly, we observed that ET induced a very fast and marked increase in the cytosolic Ca^2+^ concentration in the cultured granule cells, as determined using Fura2 (Fig. 3D). Preincubation with a medium containing no Ca^2+^ ions (10 mM ETGA, 0 M Ca^2+^) almost abolished the intracellular rise in [Ca^2+^] induced by 10^−7^ M ET (Fig. 3D). This suggests that this intracellular Ca^2+^ rise is due to influx of Ca^2+^ ions and not from mobilization of intracellular Ca^2+^-stores.

In the renal cell lines a causal relationship between cell toxicity and ET oligomerization has been proposed. Since ET oligomerization, but not ET binding, depends on the presence of cholesterol [10]–[12], we examined whether cholesterol sequestration by methyl-β-cyclodextrin interfered with the cytotoxicity of ET. Granule cell cultures were incubated with 1 mM methyl-β-cyclodextrin for 30 min at 37°C (a condition which efficiently prevents formation of ET-heptamers in membranes of renal cells [12]). Under these conditions, ET staining in granule cell cultures appeared qualitatively similar to that observed without methyl-β-cyclodextrin pre-treatment (compare panels Ai or Aii and Aiii in Fig.3) and a significant decrease in ET-induced glutamate release was observed (Fig. 3B). This pretreatment almost abolished the intracellular rise in [Ca^2+^] induced by 10^−7^ M ET (Fig. 3D). This is reminiscent of previous studies in renal cells, in which cholesterol depletion prevented the ET-induced intracellular rise in [Ca^2+^] [12]. Moreover no blebs were observed on the cultured cells submitted to ET 10^−7^ M for at least 20 min (not illustrated).

Overall these data confirm that the granule cell can be targeted and affected by ET, leading to [Ca^2+^]_i_ rise likely due to Ca^2+^ influx, glutamate release and membrane severing. Since cholesterol sequestration impairs these effects and efficiently prevents formation of ET-heptamers in membranes and cytotoxicity in renal cells [12], it is tempting to speculate that they relate to the pore forming activity of ET.

### ET directly stimulates glutamatergic, and indirectly GABAergic, synaptic transmission afferent to the Purkinje cells

To analyze the effect of ET on neurotransmission (i.e. neuronal network activity and neurotransmitter release), we used cerebellar slices cultured for 2–3 weeks *in vitro*, which form a simplified cerebellar neuronal network, and are thin enough (few layers of neurons) to allow access of ET to all the neurons present in the slice, which is not the case in acute slices. In cultured slices, Purkinje cells receive glutamatergic (excitatory) inputs originating only from granule cells while the GABAergic (inhibitory) inputs are made by inhibitory interneurons and Purkinje cells [29], [30].

**Figure 4:**
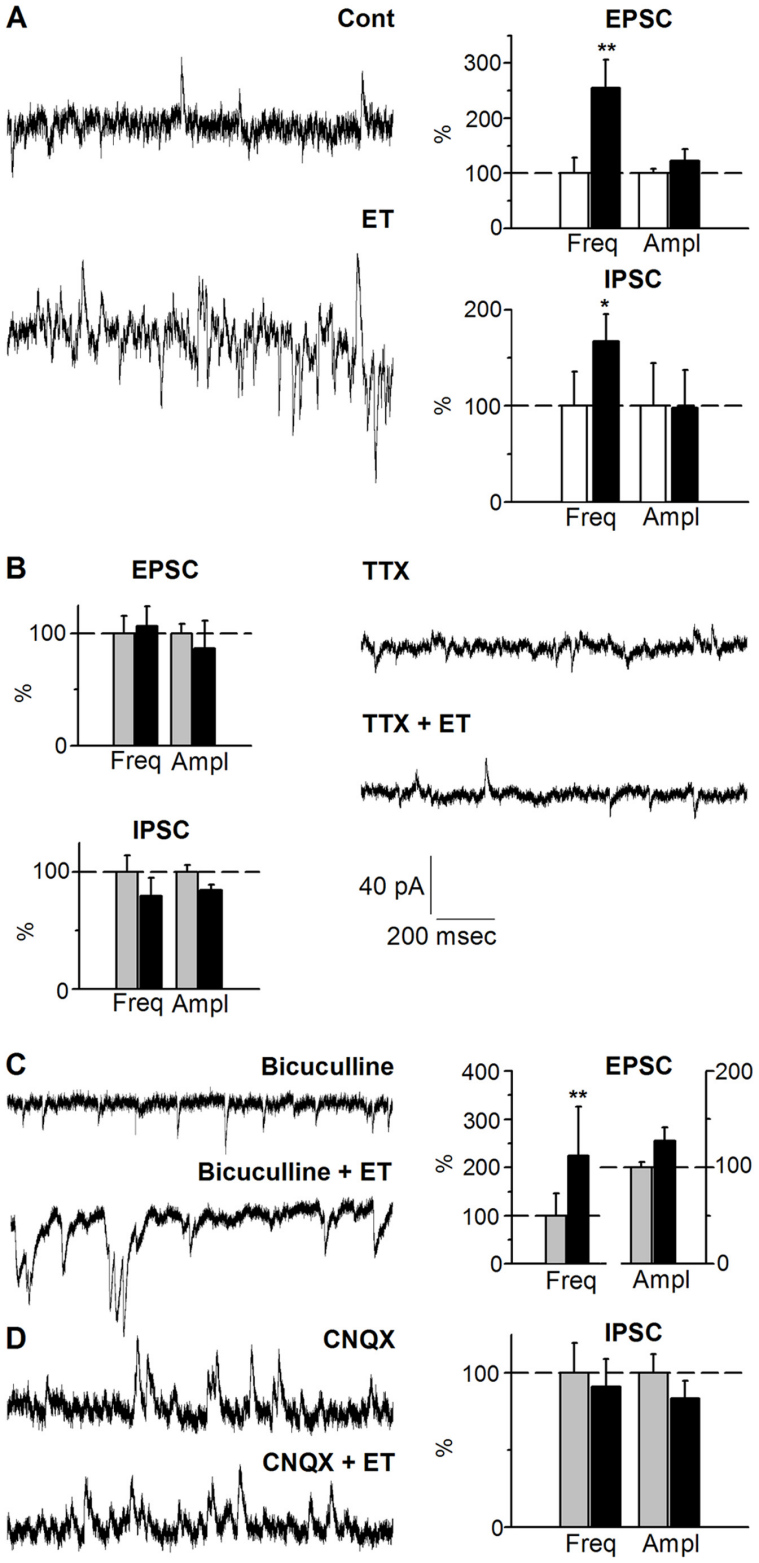
ET stimulates excitatory and inhibitory synaptic transmission onto the Purkinje cells. A, right: spontaneous PSC detected in voltage-clamped Purkinje cells maintained at −60 mV, in absence (Cont) or 5 min after ET (10^−7^ M) application. The relative mean frequencies (Freq) and amplitudes (Ampl) of spontaneous EPSC (upper graph) or IPSC (lower graph), before (white bar) or 5–7 min after 10^−7^ M ET was added (black bar), *n* = 15 distinct experiments. B–D, same kind of measurements but after pre-treatment (B) with TTX (10^−6^ M for 10 min, *n* = 18, (C) with bicuculline (10^−5^ M for 5 min) to block the IPSC (*n* = 17), or (D) CNQX (10^−5^ M for 5 min) to block the EPSC (*n* = 18). The frequencies and amplitudes are presented as percent of control condition (i.e. without any treatment, white bars) or after pre-treatment (grey bars), and after subsequent application of ET (black bars). **: *p*<0.01, *: *p*<0.05, otherwise *n.s.* Same scale for all current traces.

To allow simultaneous recording of spontaneous postsynaptic, mostly Na^+^-dependent, excitatory currents (sEPSC) resulting from vesicular release of glutamate or spontaneous postsynaptic inhibitory, Cl^−^-dependent, currents (sIPSC) due to GABA release, Purkinje cells were voltage clamped (whole cell patch configuration) at a holding potential of −55 mV (note that E_Na_^+^ ∼ +107 mV and E_Cl_- ∼−92 mV using the mentioned extracellular and intrapipette solutions). Typical postsynaptic current traces are shown in Figure 4A. sEPSC and sIPSC frequencies were determined from recording epochs of 5 min duration, made before and after ET (10^−7^ M) application. Due to the irreversible action of ET, only one Purkinje cell could be recorded per cultured slice. After a delay of a few minutes (281+/−53 s), sEPSC frequency was significantly increased by a factor of ∼2.5 fold (*p*<0.01) after ET application. Bar chart in Figure 4A summarizes data from *n* = 15 independent experiments. sIPSC frequency was also significantly increased, but only by ∼60% (*p*<0.05) (Fig. 4A). Then, during the course of each experiment both sEPSC and sIPSC frequencies declined. No change in plasma membrane resistance of the Purkinje cells was detected (control: 42.1+/−5.2 MΩ, *vs* ET: 43.4+/−4.1 MΩ, *n.s.*).

No significant change was detected in the mean amplitudes of the sEPSC and sIPSC, under control condition (cont.) and after ET application (Fig. 4A): (mean sEPSC cont.: 16.3+/−1.3 pA, *vs* ET: 20.0+/−3.5 pA, *n.s*, from *n* = 15 experiments during which at least 200 synaptic events were averaged; mean sIPSC cont.: 38.5+/−16.9 pA, *vs* ET: 37.7+/−14.8 pA, *n.s.*, averages from *n* = 15 experiments, 200 synaptic events each). The amplitude of the first and main peak in the sEPSC and sIPSC amplitude distribution histogram (not shown), which corresponds to the monoquantal postsynaptic currents, was not modified after ET (sEPSC cont.: 14.6+/−2.9 pA, *vs* ET: 15.2+/−1.9 pA; sIPSC cont.: 12.6+/−1.0 pA, *vs* ET: 14.2+/−1.9 pA, same *n* and averaging conditions as described just above). This indicated that ET does alter neither the size of the glutamate or GABA transmitter quantum (i.e. no changes in synaptic vesicle transmitter content or density in postsynaptic receptors), nor the postsynaptic properties of the Purkinje cells.

Since ET has been reported to make pores in synaptosomal membranes assumed to correspond to nerve ending membrane [11], we investigated whether stimulation of synaptic activities by ET might result from a direct effect of the toxin on the nerve endings (albeit no-significant staining was detected, see Table 1 and Figs. 1 and 2). To pharmacologically isolate the nerve endings from action potentials propagating in the neuronal network, we bath applied the potent blocker of Na^+^-channels Tetrodotoxin (TTX, 10^−6^ M for at least 15 min) prior to application of ET. On its own, TTX reduced the frequency of both the sEPSC (by 25.3+/−11.5%, *p*<0.05) and sIPSC (by 40.5+/−8.3%, *p*<0.01 leaving only the miniature EPSC and IPSC, which are due to spontaneous fusion of synaptic vesicles containing glutamate or GABA. Subsequent application of ET (10^−7^ M) did not induce significant change in respective frequencies or amplitudes of miniature EPSC or IPSC (Fig. 4B) ruling out the possibility that ET directly acts on nerve endings. This is consistent with i) the lack of significant ET-staining in the molecular layer of the cerebellar cortex which contains all granule-to-Purkinje cells synaptic contacts (see above) and ii) previous report that ET fails to trigger glutamate release from brains synaptosomes [27].

The TTX experiments suggest that the ET-stimulated increase in synaptic activity is due to an enhanced activity in the neuronal network. To discriminate between direct stimulation of ET on the excitatory neurons, from their dis-inhibition following alteration of inhibitory transmission, we examined the synaptic effects of ET after pharmacological isolation of glutamatergic or GABAergic transmission, using either the GABA-receptor antagonist bicuculline, or CNQX to block glutamate receptors of AMPA-type. Bath application of bicuculline (10^−5^ M, bicu) on the cultured slices abolished the occurrence of all the sIPSC detected in the Purkinje cells (Fig. 4C) and, due to dis-inhibition of glutamatergic neurons, this also potentiated the frequency of sEPSC (to 327+/−151%, *p*<0.01). Subsequent application of ET (still in the presence of bicuculline) led to a further potentiation of the sEPSC frequency by a factor ∼2.5, *p*<0.01, (Fig. 4C) with no significant changes in the mean sEPSC amplitude (mean sEPSC bicu: 12.7+/−0.7 pA, *vs* bicu + ET: 16.2+/−1.8 pA, *n.s.*) or monoquantal sEPSC (first peak sEPSC bicu: 10.8+/−0.5 pA, *vs* bicu + ET: 12.1+/−0.9 pA, *n.s.*). When CNQX (10^−5^ M) was applied, sIPSC frequency was reduced (to 62.2+/−12.2%, *p*<0.01) consistent with the fact that, in the slices, the firing activity of the inhibitory interneurons is driven by the excitatory inputs they receive [29], [30]. Subsequent application of ET (10^−7^ M) failed to induce any increase in the sIPSC frequency recorded in the Purkinje cells (Fig. 4D) with no significant changes in the mean sIPSC amplitude (mean sIPSC-CNQX: 17.5+/−2.2 pA, *vs* CNQX + ET: 14.6+/−1.9 pA, *n.s.*) or monoquantal sIPSC amplitude (first peak sIPSC-CNQX: 12.9+/−1.0 pA, *vs* CNQX + ET: 11.6+/−0.8 pA, *n.s.*). This important finding unambiguously indicates that ET directly and selectively stimulates glutamatergic transmission by the granule cells, which are the only glutamatergic neuronal cell type in cultured cerebellar slices. Moreover, a lack of ET effect on the GABAergic transmission, when ET is applied after excitatory transmission has been blocked, indicates that the stimulation of the inhibitory activity by ET, as observed in absence of pretreatment (Fig. 4A), is indirect and results from enhanced GABAergic neurons firing activity in response to increased firing activity of the granule cells.

### ET directly depolarizes granule cell somata causing their firing

**Figure 5:**
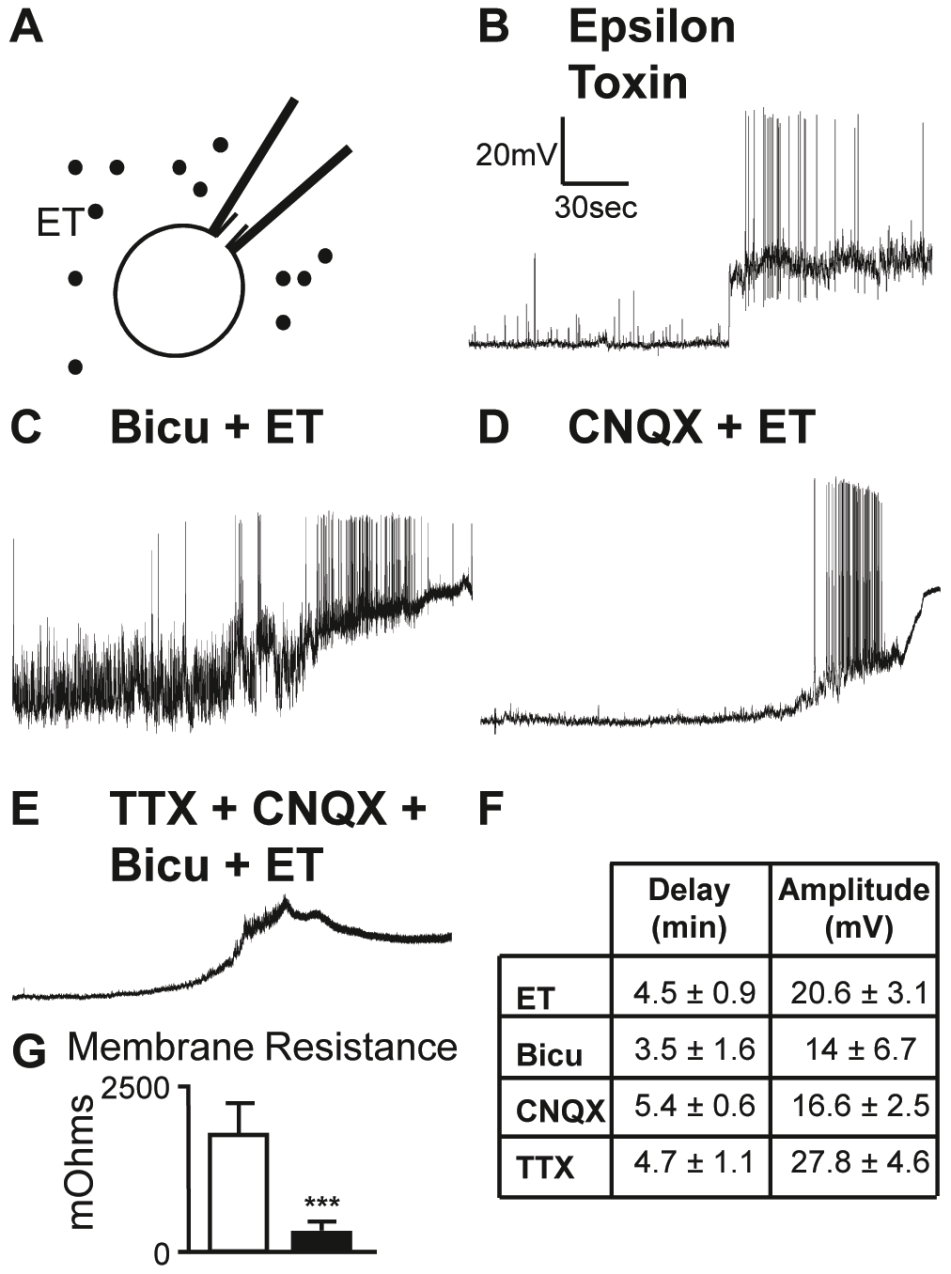
ET depolarizes granule cells in cultured slices. A, Schematic representation of the recording configuration (Whole Cell). B–E, typical membrane potential changes recorded in granule cells (using the Current Clamp mode) adjusted at −60 mV, after application of 10^−7^ M ET but without (B, *n* = 15) or after pre-treatment for 10 min with (C, *n* = 6) Bicuculline (Bicu, 10^−5^ M), (D, *n* = 7) CNQX (10^−5^ M) or, (E, *n* = 8) a cocktail of Bicuculline (10^−5^ M), CNQX (10^−5^ M) and TTX (10^−6^ M). F, quantification of the delay and amplitude of the depolarization induced by ET. For the corresponding *n*, see above. All comparisons *vs* ET alone are *n.s.* G, Changes in membrane resistance of the granule cells before (white bar) and 5 min after 10^−7^ M ET (black bar) (*n* = 15, *p<*0.001). Same scale for all voltage traces.

The above immunostaining data and our electrophysiological findings clearly pinpoint the granule cells of the cerebellar cortex as a major ET target. Since ET binds to granule cell somata (Table 1, Fig. 2) and has effects on them in primary culture (see above, Fig. 3), we examined whether and how ET acts on the cell body of granule cells. Granule cells were current-clamped under the whole cell patch configuration (Fig. 5A). The transmembrane potential changes were continuously monitored, during a control resting period (at least 15 min) and after ET was bath applied. In all of the *n* = 15 experiments performed (one cell recorded per slice), after a delay of 3–5 min (see Table enclosed in Fig. 5F), ET induced a ∼20 mV depolarization of the granule cell leading to bursts of action potentials (Fig. 5B) followed by a plateau depolarization in about 2/3 of the cells.

We could not completely exclude the possibility that the ET-induced depolarization of the granule cell soma was indirect, mediated by an ET-induced release of excitatory neuroactive molecules from neighboring glial cells (as the oligodendrocytes that are targeted by ET). Thus, we repeated the above described experiments after the recorded granule cells were pharmacologically isolated by pre-incubating the slices with agents aimed at blocking receptors to the neurotransmitters or reducing the firing activity. Application of bicuculline (10^−5^ M, for 10 min, see a typical recording from a series of 6 in Fig. 5C), CNQX (10^−5^ M, for 10 min, see a typical recording from a series of 7 in Fig. 5D), TTX alone (10^−6^ M, for 10 min, not illustrated) or a mixture of the 3 drugs (see a typical recording from a series of 8 in Fig. 5E) did not prevent the depolarizing effect of ET on granule cells (Fig. 5F).

When determined 1 min after the onset of ET-induced depolarization, the membrane resistance was found about 8 fold decreased (from about 2 GigaΩ to about 270 MegaΩ, *p*<0.001; see Fig. 5G). Note however that the residual membrane resistance was still high, suggesting that no membrane disruption occurs. These findings bring rational for explaining how TTX suppressed the stimulation of glutamate release by ET: TTX, which does not interfere with the initial depolarizing effect of ET on granule cells, prevents propagation of its consequences (i.e. the action potentials) to nerve endings.

### Characterization of the neuronal membrane changes induced by ET

**Figure 6:**
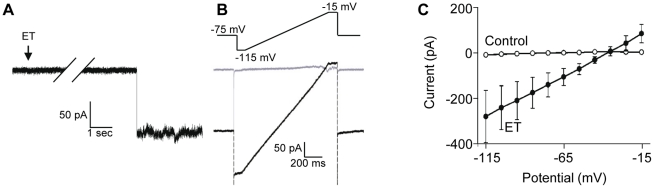
Membrane current induced by ET in granule cells. Granule cells were maintained under voltage clamp using the whole cell configuration. A, a recording taken from a series of 15 independent experiments (granule cells hold at −75 mV) during which slices were preincubated for 10 min with TTX (10^−5^ M), TEA (1 mM), 4-AP (2 mM), CNQX (10^−5^ M) and bicuculline (10^−5^ M) before application of ET (10^−7^ M, arrow). Note the abrupt large inward current step that manifests action of ET on the membrane characteristics. B, During the course of the same series of experiments, membrane holding potential was changed from −75 mV to −115 mV, followed by depolarizing ramps from −115 mV to −15 mV, before returning to −75 mV. This paradigm was performed before application of ET and after the toxin had induced an abrupt change in the whole cell current, as illustrated in A. Typical currents (before: grey; after ET: black) are shown. C) Currents traces were pooled under control (before ET) or after ET and averaged, to build the I =  f(V) relationship.

We investigated the mechanisms by which ET affects neuronal plasma membrane. To this aim we recorded the transmembrane current (voltage-clamp, whole cell configuration) changes produced by ET on granule cells maintained at –75 mV. To minimize the contribution to the whole cell membrane current of the recruitment of endogenous voltage- or neurotransmitter gated channels, the preparations were preincubated for 10 min with a cocktail containing bicuculline (10^−5^ M), CNQX (10^−5^ M), TTX (10^−6^ M), TEA (2 mM) and 4-aminopyridine (1 mM), these two latter drugs blocking endogenous K^+^-selective voltage-dependent channels. Under this experimental configuration, the ET effect on granule cells was revealed by abrupt inward current changes that may reach ∼100 pA amplitude, during which smaller steps of ∼15 pA could be also observed. Such current changes (see the downward step in the current trace illustrated in Fig. 6A) appeared after an averaged delay of 5±1.5 min, very similar to the delay observed for ET-induced depolarization of granule cells (Fig. 5F). The transmembrane current increased with time either progressing by large or smaller steps (not illustrated). On average, as determined within 1 min after the first membrane current change was detected, the increase in plasma transmembrane current due to ET was ∼50 pA (holding potential of –75 mV), but it increased with time. These findings suggest that the ET-induced transmembrane current is contributed by activation/opening of several pores or channels, the recruitment of which increases with time.

To determine the reversal potential of the ET-induced current, we built the current (I) to membrane potential (V) relationship (i.e. the I =  f(V) curves). Given the kinetic of the membranes changes caused by ET, we could not construct the I =  f(V) curves by analyzing the current traces corresponding to voltage square pulses of incremented amplitude, but, instead, we used a continuous depolarizing ramp of membrane potential (from −115 mV to −15 mV, 100 mV.s^−1^). The corresponding ramp currents increased almost linearly with transmembrane potential (see example in Fig. 6B). Figure 6C shows the average I =  f(V) curve (summary of 5 experiments, data denoted by filled circle). The I =  f(V) curve showed no rectification between −115 and −15 mV, and the reversal potential for ET-induced current was ∼−25 mV.

**Figure 7:**
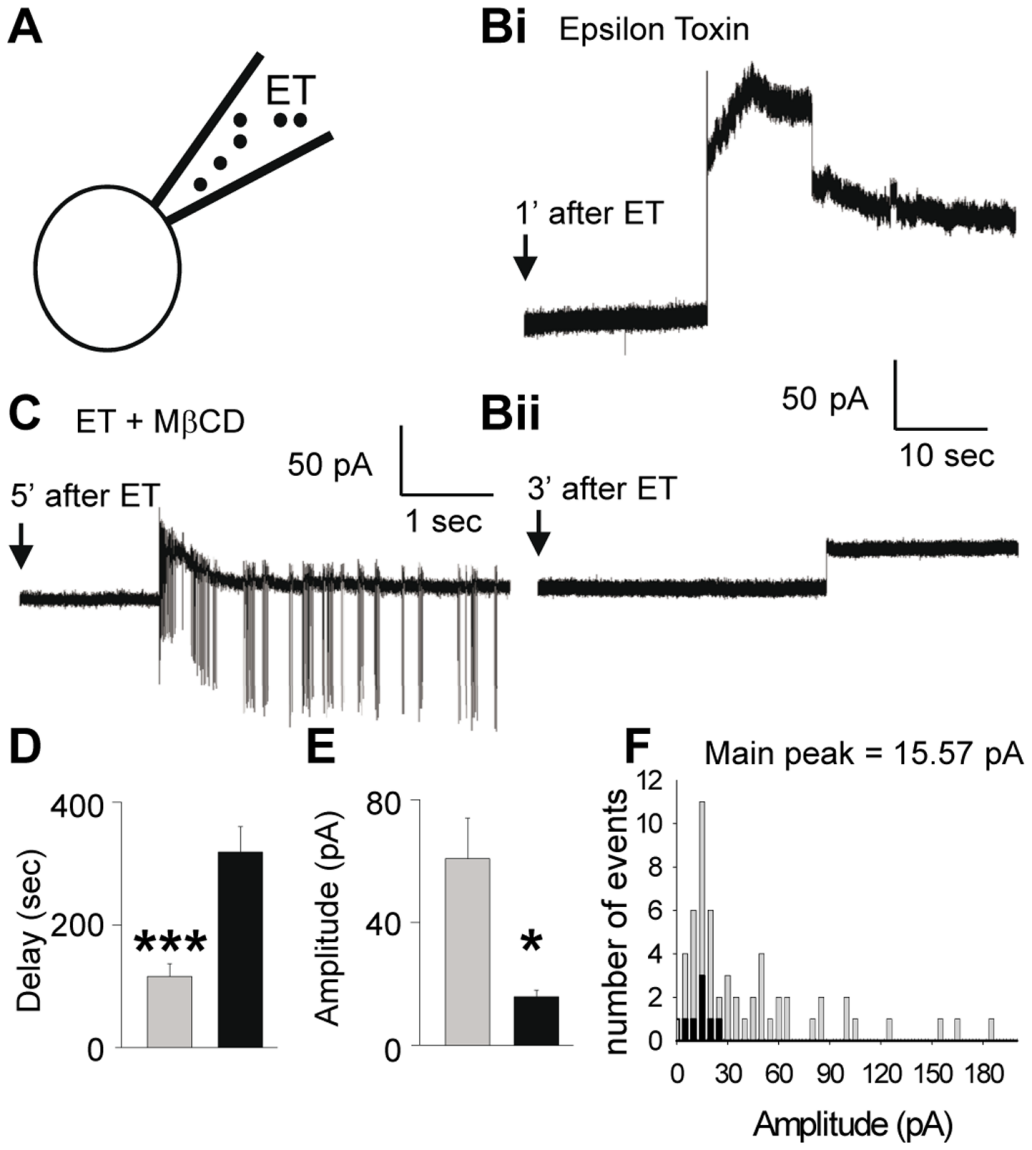
Abrupt current changes induced by ET in membrane patches. A, Schematic representation of the recording configuration (Cell attached). ET (10^−7^ M) was applied inside the patch-pipette. B–C, typical membrane current changes recorded after sealing the patch-pipette onto a granule cell membrane (membrane potential maintained at −45 mV using the Voltage Clamp mode), without pre-treatment (B_i_, B_ii_) or after pre-treatment (C) for 30 min with 1 mM of methyl-β-cyclodextrin (MβCD). The corresponding average delays (D) before current changes were detected, and average amplitude of the detected current changes (E), and distribution amplitude (F) of the observed current changes. Grey and black bars denote experiments performed using ET alone (mean from 34 recordings) or after pre-treatment with MβCD (mean from 16 recordings) respectively.

We also attempted to investigate the elementary membrane changes induced by ET. In a series of *n* = 100 experiments, ET (10^−7^ M) was applied inside the recording pipette prior to cell-attach recordings were made (scheme in Fig. 7A). To minimize leakage of ET from the tip of the micropipette during the micro-positioning, a negative pressure was applied. The pipette potential was maintained at 0 mV. In 34 out of the 100 experiments performed, within the few minutes after the sealing of the tip of the patch pipette was established onto the outer face of the plasma membrane of the granule cells, we observed the appearance of abrupt increases (i.e. step) in the membrane current (Fig. 7B). Their amplitude distribution showed a main peak at ∼16 pA, but the average amplitude was 70 pA (Fig. 7E). When control recordings (for more than 20 min without ET inside the micropipette) were performed, no similar change in transmembrane current was observed (occurrence of abrupt membrane current change: + ET, *n* = 34/100, - ET: *n* = 0/26, *p*<0.001). This finding revealed a direct effect of ET on the granule cell membrane. In the 66 negative experiments, it was unclear whether ET had failed to produce an effect (may be no ET receptor was present in the recorded patch of membrane or the pore/channel activation occurred during the process of sealing of the patch pipette to the plasma membrane of granule cell).

Since heptamerization of ET and ensuing pore forming activity needs the presence of cholesterol [11], [12], we examined whether the membrane effects of ET were sensitive to methyl-β-cyclodextrin. Slices were preincubated with methyl-β-cyclodextrin (1 mM for at least 20 min at 37°C), and the recording was performed with micropipettes containing ET (10^−7^ M) + methyl-β-cyclodextrin (1 mM). Surprisingly, in 16 out of 39 experiments (i.e. 41%, similar as the 34% reported above in methyl-β-cyclodextrin untreated slices), ET was still able to induce outward membrane current steps (a typical example is provided in Fig. 7C). No occurrence of abrupt membrane current change was observed in absence of ET (+ MβCD and + ET, *n* = 16/39, + MβCD - ET: *n* = 0/21; *p*<0.001). However, cholesterol sequestration increased the delay before observing the ET-induced outward current steps (Fig. 7D), and diminished their average amplitude (Fig. 7E). Despite the number of recordings made after methyl-β-cyclodextrin pre-treatment was fewer than in control, the main peak in the distribution amplitude histogram of the ET-induced step currents was unchanged (Fig. 7F, peak at around 16 pA).

## Discussion

Following acute injection of ET into animals [20], excessive neurotransmitter release has been reported. This has been proposed to result from ET-induced brain micro-vessels lesions [27], [31], ensuing diffuse brain edema, and leakage of transmitters from damaged neurons [4]. Albeit some biochemical studies performed on rat brain synaptosomes have suggested a direct action of ET on neurons [11], [14], [26], the more recent binding studies performed using GFP-tagged ET, have disputed this possibility. Indeed only the astrocytes and myelin (i.e. oligodendrocytes), microglial cells, but not neurons, were reported to be targeted by GFP-tagged ET [25], [27]. In this study, we bring a set of compelling evidence (immunolabeling, functional data) that refutes part of the previous deductions and establishes unambiguously that ET can directly act on a subset of neurons, the granule cells in the cerebellar cortex. The reason for the discrepancy between our data and previous reports, may relate to i) unknown properties of the GFP-tagged ET and/or, ii) the fact that the staining of the neurons was faint as compared to that of the oligodendrocytes, hence difficult to detect.

### ET binds to oligodendrocytes but not to astrocytes

The lack of ET-staining on the molecular layer and on GFAP-expressing cells in the granule layer of acute cerebellar slices or primary cultures from the mouse cerebellar cortex indicates that neither Bergman's radial glial cells nor cerebellar velimentous astrocytes are targeted by ET. This contradicts previous reports that GFP-tagged ET binds to astrocytes [25]. Maybe, the presence of the GFP-tag has miss-targeted ET in these previous studies, or the close apposition of astrocytic processes (GFAP positive) with the capillary blood vessel lead to the appearance that ET-bound to the astrocytes. However, from our present results, the possibility that a sub-population of GFAP-expressing cells may bind ET in other brain regions cannot be ruled out.

The identification of oligodendrocytes as a major ET target is consistent with a previous report showing that GFP-tagged ET binds to myelin [27], and staining of brain white matter by ET (this study). The strong staining intensity of white matter, which contains myelinated axons and oligodendrocytes cell bodies, as compared to the granular layer, suggests a greater avidity of the oligodendrocytes for ET than the granule cells. Perhaps this does relate on the high cholesterol content of their plasma membrane and of myelin [32]. Analysis of the functional consequences of ET binding on oligodendrocytes was beyond the aim of this report, but this deserves to be investigated in further studies.

### Induction of glutamate release by ET

Our observations that ET stimulates glutamate release in cerebellar primary and cultured slices are fully consistent with the several previous studies, which have shown ET-induced release of various neurotransmitters, including glutamate [19]–[21]. Since neuron and glial cells contain and can release glutamate, the large efflux of glutamate reported in poisoning experiments may have a neuronal or glial origin. Our present observation that ET directly acts on neurons, induces glutamate release in primary cultures of granule cells, and stimulates glutamatergic transmission at the granule cell - Purkinje cell synapse in slices largely supports the possibility that the release of glutamate reported in brain [19]–[21] is mostly neuronal. However, since oligodendrocytes are labeled by GFP-ET [27] as well as native ET (this study), the possibility they are a source for ET-induced release glutamate cannot be ruled out.

ET-induced release of glutamate may involve two distinct effects of ET: i) stimulation of the neurotransmitter release machinery, and ii) leakage from severed cells. Since glutamate is present in the cytosolic compartment of any kind of cells, ET-induced plasma membrane severing of the toxin's targets can lead to an efflux of glutamate. This possibility is supported by our observations that LDH, which is a cytosolic protein, is released upon application of high ET concentration (10^−6^ M). We also observed that a similar fraction of glutamate efflux induced by ET in primary culture is Ca^2+^-independent and resistant to pretreatment with methyl-β-cyclodextrin. However, our study cannot distinguish between plasma membrane disruption and formation of ET-pores large enough to allow transmembrane passage of proteins or amino acids. Since we observed that ET depolarizes neurons and induces intracellular Ca^2+^-rise, most of the glutamate efflux induced by ET at 10^−7^M may result from stimulation of the vesicular release machinery in cultured granule cells. This is supported by observations that i) when Ca^2+^ ions are lacking in the extracellular medium, ET binds to neuronal membrane but fails to induce glutamate efflux, and ii) pretreatment with methyl-β-cyclodextrin prevents induction by 10^−7^ M ET of Ca^2+^-intracellular rise as well as glutamate release. Further experiments directly altering the vesicular release machinery should be performed to confirm the vesicular origin of the glutamate released by cultured cells submitted to ET. In next paragraph, we discuss the possibility that glutamate release is a secondary, distant, manifestation of the ET effects.

### Propagation of ET-effect from its binding site to nerve endings and non-target neurons

Our labeling and functional data in nerve tissue revealed that the ET effects comprise primary action(s) close to the toxin's binding site, and secondary actions, possibly occurring distant from the binding site. Although one of the most prominent effects of ET in nerve tissue is the stimulation of glutamate release (see electrophysiological data in Fig. 4), no experimental evidence supports the possibility that ET directly acts on nerve ending. Indeed i) the molecular layer of the cerebellar cortex, which contains the glutamatergic varicosities making synaptic contacts between granule cells (highly susceptible to ET), and the dendritic tree of Purkinje cells appears almost unlabelled by ET; ii) no co-staining was observed between ET and synaptic markers such as synaptotagmin, synaptophysin and PSD-95 in the granular layer. Thus our labeling data contradict the initial reports that ET forms pores into membranes isolated from rat brain synaptosomes [11], [14] but confirms previous observations that ET-GFP fails to directly induce glutamate release from synaptosomes [27]. Consistent with the binding of ET on granule cell pericaryon, the electrophysiological measurements performed on granule cells showed that the membrane resistance is lowered by a factor ∼8 following ET application. The pore-forming activity recorded during cell-attach experiments on granule cell somata indicates that the membrane resistance change is likely to represent a primary effect of ET. This change is associated with an inward increase of the resting current in the range of 50–100 pA, which depolarizes the granule cells thus reaching the threshold for initiating action potentials. The latter propagate toward nerve endings and produces a secondary effect of ET consisting in stimulating vesicular glutamate release. This view is fully supported by our observation that TTX, which prevents action potential propagation, abolishes the stimulatory effect of ET on neurotransmitter release. Our findings are also consistent with a previous report that riluzole, which blocks persistent Na^+^- and Ca^2+^-currents [33] and thus impairs the action potential propagation, attenuates ET-induced glutamate release in the mouse hippocampus [20].

Propagation of ET effect in the neuronal network may also explain its stimulatory action on the release of other neurotransmitters (as GABA in the cerebellar cortex). In the cerebellar cortex, we did not identify neurons other than granule cells potentially sensitive to ET. In the molecular layer and the granular layer ET does bind neither to GABAergic interneurons nor to Purkinje cells. Moreover we did not detect significant change in the membrane input resistance of the Purkinje cells in presence of ET. The pharmacological dissection of the synaptic effects of ET on neurotransmission clearly indicates that the stimulation of GABA-release by ET is only indirect and results from the ET-induced increase in the excitatory activities in the neuronal network.

### Comparison of the primary effects of ET on neurons and on other cell targets

Few cell types have been identified to be targeted by ET. This includes the cells of the distal renal tubule [24], [34] and related cell lines [8], [12], [35], vascular capillary endothelial cells [23]–[25], enteric epithelial cells [2], the oligodendrocytes and peripheral Schwann cells that both contribute to neuronal axon myelination [27], specific neurons, as the cerebellar granule cells (this study), macrophages and the related brain microglial cells [27], [36]. This strongly suggests that this biological spectrum of selectivity relates to the expression of yet unidentified specific receptor(s) by ET cell targets.

With regard to the other aspects of the cellular and molecular effects of ET, most of the current knowledge has been established using renal cells and comparison of the data established using neurons (this study) and renal cells reveals several commonalities. For example the extent of ET binding on the baso-lateral and apical membranes of renal cells differs [12], and in the acute cerebellar slices, where granule cells are highly differentiated, ET labels their cell body but not axons and nerve terminals. This suggests that only specific membrane domains allow ET binding as revealed by immunostaining. Further experiments are needed to determine whether this results from a local enrichment in ET receptors or/and organization of phospholipids and cholesterol micro-domains. In renal cells or granule neurons, the data suggest that part of the ET action, but not ET binding, relies on cholesterol or integrity of cholesterol containing microdomains: pretreatment with methyl-β-cyclodextrin abolishes ET-induced Ca^2+^-rise in granule cell primary cultures (this study) as well as in renal cells [12]. This is less clear with regard to the pore forming action of ET: it is abolished in renal cells when pre-treating the cells with methyl-β-cyclodextrin [8]–[12], but in granule neurons the ET-induced changes in transmembrane currents were not abolished in presence of methyl-β-cyclodextrin (cells pretreated for 30 min); yet these currents were slightly delayed and their mean amplitude lowered but without affecting the main peak of their distribution amplitude. Thus the question arises of whether the observed ET-induced abrupt changes in membrane current relate to the pore-forming activity of ET or to activation of endogenous conductances. Although we cannot exclude this later possibility, we have to point the facts that ET has been reported to form pores in liposomes containing phosphatidylcholine exclusively [10] and that pre-treatment with 1 mM methyl-β-cyclodextrin for 30 min might not be sufficient to extract enough cholesterol from the granule cell membranes, as discussed elsewhere [37]. This makes the possibility of endogenous conductance activation less likely.

### The pore formed or activated by ET in neuronal membrane

In membrane bilayers, ET pore has been previously determined as a general diffusion pore, allowing Cl^−^, K^+^ Na^+^ ions to flow, but with a preferential anionic selectivity (permeability ratio P_K+_/P_Cl_- ∼0.30) [9]. Thus, if P_Na+_ ∼ P_K+_, and given E_K_+  =  −101 mV, E_Na_+  =  +107 mV, and E_Cl_−  =  −92 mV, under our experimental conditions the expected equilibrium potential should be ∼ −37 mV (based on the Goldman-Hodgkin-Katz equations). Our findings that the reversal potential for ET-current was ∼ −25 mV, indicates that the ionic selectivity determined previously using bilayers [9] does not fully apply to pores formed or activated into neuronal membrane. May be, the ET- induced intracellular Ca^2+^ rise (which is abolished when cells are incubated with no extracellular Ca^2+^ ions) is due to Ca^2+^ permeation through ET formed pores, thus explaining a more depolarized equilibrium potential than that expected. Given the depolarizing effect of ET on granule cells, further experiments are needed to determine the respective contribution of ET-pores and endogenous voltage-dependent Ca^2+^-channels in the ET-induced rise of intracellular Ca^2+^.

Considering that the resting potential of granule cells submitted to cell-attached recording is ∼−65 to −60 mV and the reversal potential for ET-induced current ∼ −25 mV, the ET-induced current steps of 15 pA (see main peak of the distribution, Fig. 7F) may correspond to the opening of single state conductance large pores of over 370 pS, as determined using the Ohm's law. This is far larger than the conductance of endogenous neuronal channels and reminiscent of that determined for ET-pore in lipid bilayers (550 pS in presence of 1 M KCl) [9]. Using the membrane model where pores are analog to parallel resistors, the large fall in membrane resistance that we observed in granule cells (from ∼2 GΩ to ∼270 MΩ) can be accounted by formation of ∼9 pores per granule cell.

To conclude, the emerging picture from our labeling and functional studies is that granule cells and oligodendrocytes are major ET targets in the mouse cerebellar cortex. In view of the high bio-hazard of ET [38], identification of neurons as ET targets shed a new light on the mechanisms by which ET can exert deleterious action on the central nervous system. It probably results from a direct severing action of ET on subsets of neurons (our study), and indirect damage resulting from vasogenic edemas [4]. The direct effect of ET on neurons is likely to amplify the highly potent systemic action of the toxin, and this may explain why ET lethal activity is 100-fold higher than that of other structurally related pore-forming toxins [39].

## Materials and Methods

### Ethic Statement

All experiments have been conducted using protocols designed according to the European and French guidelines on animal experimentation and approved by the direction of the Bas-Rhin veterinary office, Strasbourg, France; authorization number 67-295 to JdB), and the direction of Paris veterinary office, France, (authorization number 75-279 to MP).

### Epsilon toxin materials

ET was purified from an overnight culture of *C. perfringens* type D strain NCTC2062 as previously described [8]. The purity of ET (>90%) was checked by SDS-PAGE electrophoresis. Unless otherwise stated, ET was extemporaneously diluted to a final concentration of 10^−7^ M in the superfusion medium from stock aliquots stored at −80°C. Specific anti-ET antibodies were raised in rabbit with formalin treated wild type epsilon toxin as described previously for other clostridial toxins [40]. Antibodies against epsilon toxin were purified from hyper-immune rabbit sera using wild type epsilon toxin coupled to cyanogen bromide activated sepharose 4B (Amersham Biosciences) according to the manufacturer's recommendations. Specificity for ET of immunoaffinity purified antibodies was checked by immunoblotting experiments: crude culture supernatant of *C. perfringens* type D strain NCTC2062 (100 µg proteins) was run on a SDS-PAGE (10%), and then transferred on nitrocellulose. The membrane was blocked with non-fat milk and incubated with rabbit anti-epsilon toxin (1∶2000) and subsequently with protein A-peroxidase. Protein bands were visualized using the ECL detection system (InVitrogen, Gergy-Pontoise, France).

### Whole brain and cerebellar preparations

Acute slices of whole brain or cerebellum alone were prepared according to standard procedures. 150 µm thick parasagittal cerebellar slices from 25–30 days old C57/BL6 mice were made following a protocol approved by the European and French guidelines on animal experimentation. Briefly, mice were killed by decapitation under isoflurane general anesthesia (AErrane, Baxter SA, Maurepas, France; Isoflurane anesthesia unit from Medical Supply Service lnt, England). Whole brains or only cerebella were dissected in ice-cold physiological medium (i.e. artificial cerebrospinal fluid) equilibrated with 95% O_2_ and 5% CO_2_, and containing: NaCl 124 mM, KCl 2.7 mM, CaCl_2_ 2 mM, MgCl_2_ 1.3 mM, NaHCO_3_ 26 mM, NaH_3_PO_4_ 0.4 mM, glucose 18 mM and ascorbate 4 mM. Slices were made using a Leica VT1000S slicer, and then maintained at the desired temperature in the same physiological solution.

Cerebellar slices in organotypic culture were prepared from cerebella removed from 7–10 day old C57/BL6 mice and cultured using the roller tube technique modified from [41] as described previously [29], [42]. In short, parasagittal cerebellar slices (350 µm thick) cut under aseptic condition were embedded in clotted chicken plasma on glass cover-slips and placed in culture tubes containing 750 µl of culture medium made of 25% heat-inactivated horse serum, 50% Eagle's basal medium, 25% HBSS supplied with 33.3 mM D-glucose and 0.1 mM glutamine. The tubes were placed in a roller drum at 36°C. To delay the overgrowth of macrophages, glial cells and fibroblasts in the cultured slices, 2 days after the culture was started, uridine, cytosine-β-D-arabino-furanoside and 5-fluorodeoxyuridine (Sigma) were added (10^−7^ M final) to the culture medium for 24 hours. The cultures were fed once a week by renewing the culture medium.

Cerebellar cells primary cultures highly enriched in granule cells were prepared from 7 day old C57BL6 mice. In brief, dissected cerebella were transferred in Hank's buffer salt solution (HBSS). Enzymatic dissociation of the cells was made by incubating cerebella for 5 min at 37°C in trypsin 0.05%-EDTA 0.02% solution (GIBCO), then in papaïn (Sigma-Aldrich) 15 U/ml during 20 min at 37°C. The enzymatic reaction was stopped by adding B27/Neurobasal medium (GIBCO). After centrifugation (1 min, 120 g), the pellet was resuspended in Neurobasal medium and mechanically dissociated. Cells were plated on poly-ornithin-coated glass coverslips and cultured in modified Neurobasal medium. To increase granule cells survival the culture medium was supplemented with KCl 30 mM, insulin 1 µM, putrescin 100 µM, progesterone 20 nM, transferin 100 µg/ml, and sodium selenite 30 nM. Experiments were performed after 5 days in vitro (5 DIV).

For glutamate release studies, cells were plated on poly-L-lysine-coated (10 µg/ml) 24-well Corning dishes (3–5 10^5^ cells in 30 µl) and then incubated at 37°C in a water saturated atmosphere of 5% CO_2_-air.

### Immunodetection of ET and immunocytochemistry of specific cell markers

Whole brain or cerebellar acute slices (150 µm thick) were pre incubated with BSA (1% in physiological medium), then submitted to ET (at the concentrations and for the indicated duration at room temperature) and then fixed using paraformaldehyde 4% in PBS at 4°C overnight. Cerebellar cells primary cultures or, when needed, slices were fixed in paraformaldehyde 4% in working solution during 20 min before incubation with ET. Fixed slices were permeabilized using Triton 0.1% for 1 day followed by 3 washes in PBS. Then, the fixed slices were transferred in a working solution containing PBS with 1% bovine serum albumin +10% normal goat serum (Chemicon-Millipore) for 2 h to saturate non-specific sites.

To reveal the labeling contributed by ET, the primary anti-toxin antibodies (see above) were applied (dilution 1/150) overnight at 4°C. Secondary anti-rabbit antibody tagged with Alexa 488 or Alexa 546 (Molecular Probes, 1/2000) was applied overnight at 4°C. Whole brain or cerebellar slices were then mounted on glass coverslips with mowiol (Calbiochem).

To identify the cells targeted by the toxin, a similar protocol was applied except that the immuno-labeling against ET and the cell marker of interest were performed simultaneously by applying the primary antibodies overnight at 4°C (ET: antibodies from rabbit; cell marker: antibodies from mouse, except when specified) followed by 3 washes in the working solution, by overnight application of the secondary antibodies tagged with Alexa 546 (Molecular Probes, diluted 1/2000), at 4°C. When the primary anti-cell marker antibodies were from rabbit, we used the two steps sequential process described previously [43]: tissue preparations were first treated for ET immunodetection and then, after a 20 min post-fixation in paraformaldehyde 4%, the preparations were processed for cell marker detection.

The following primary antibodies were used: Glial Fibrillary Acidic Protein (GFAP) (Sigma-Aldrich, mouse monoclonal diluted 1/400), 2′,3′-Cyclic Nucleotide 3′-Phosphodiesterase (CNPase) (Sigma-Aldrich, mouse monoclonal diluted 1/1000), synaptotagmin (1D12 mouse monoclonal, diluted 1/500, a generous gift from Dr. M. Seagar, Marseille, France), alpha-6-GABA_A_ receptor subunit (Chemicon-Millipore, rabbit polyclonal diluted 1/1000), Kv3.1b (Sigma-Aldrich, rabbit polyclonal, diluted 1/200), parvalbumin (Sigma-Aldrich, mouse monoclonal clone PARV-19, diluted 1/1500), MAP-2 (Sigma-Aldrich, mouse monoclonal, diluted 1/250), Post-Synaptic Density Protein 95 (PSD-95; Chemicon-Millipore, mouse monoclonal, clone 666-1C9, diluted 1/100), calbindin (Sigma-Aldrich, mouse monoclonal clone CB-955, diluted 1/1500)

When needed after the immunostaining process the cell nuclei were stained using the DNA marker DRAQ5™ (Biostatus Ltd, UK), 10 µM final in PBS.

### Imaging and co-staining analysis

Unless otherwise stated, confocal images (confocal microscope Zeiss LSM 510, software release 3.2; fields of 1024×1024 pixels, magnification x40, initial pixel size of 0.053 µm^2^) were acquired using 500–530 nm (green, channel 1), 560–615 nm (red, channel 2) and >633 nm (ultrared for DRAQ5) filters at the Imaging Facility of the Institut Fédératif des Neurosciences (IFR37) at Strasbourg (France). The possible presence of ET on a specific cell type (identified on the basis of an immuno-labeling against a marker of interest) was tested: if ET binds to a cell type expressing a specific marker of interest, the respective intensities of the corresponding fluorescent immuno-signals should be positively correlated. To examine this issue, regions of interest (ROI; e.g. cerebellar molecular layer, granular layer, white matter) of at least 100,000 pixels were determined under each of the studied conditions and taken from at least triplicates of the immune-labeling experiments. For each ROI, an intensity threshold was applied (10% of maximum amplitude for each channel) for either channel 1 or 2. Then for each channel, the signal intensity was normalized to the maximum observed in the ROI [44] and the Pearson's correlation coefficient (*r_p_*) was determined for channels 1 and 2.

### Calcium imaging

Intracellular calcium changes were monitored using the Fura-2 probe (Molecular probes) as described [45]. Cerebellar primary cultures enriched in granule cells were incubated in the presence of Fura-2-AM + pluronic acid (2 µM each) for 30 min at 37°C. Cells were then washed for 20 min in a solution containing NaCl 130 mM, KCl 5.4 mM, CaCl_2_ 2 mM, MgCl_2_ 1 mM, D-glucose 5.5 mM, Hepes 10 mM, adjusted at pH 7.2 and placed in an inverted microscope (Axiovert, Zeiss). They were alternatively illuminated at 350 nm and 380 nm and images pairs were recorded using an EM-CCD camera (Hamamatsu) at 520 nm every 5 s for 20 min. The ratio of the fluorescence intensities (Ex 350 nm/Ex 380 nm) was calculated on a pixel basis for each image pair using the Metafluor software (Molecular Devices). The mean value of the fluorescence ratio was normalized for each region of interest and the intracellular [Ca^2+^] changes were estimated without further calibration.

### Electrophysiological recordings

Organotypic cerebellar slices cultured for 2 weeks were placed in a recording chamber fixed on the stage of an upright microscope (Nikon Eclipse FN1 or Olympus BX51WI). The recording chamber was filled with a physiological solution containing in mM: NaCl 145, KCl 2.7, CaCl_2_ 5, MgCl_2_ 0.5, Hepes 1, glucose 5.6, pH 7.4 adjusted with TrisOH. Neurons to be recorded were identified at the 40x magnification under DIC. Purkinje cells and granule cells were identified by their typical morphology: in cultured slices, Purkinje cells have large cell body (15–20 µm) characterized by highly refringent nucleolus, whereas granule cell are small (5–8 µm) and mostly spherical.

Whole-cell patch clamp recordings were carried out at room temperature using an Axopatch 200A Amplifier (Axon Instruments), under current- or voltage-clamp modes. Electrodes of 5 MΩ (for Purkinje cells recording) or 15 MΩ (for Granule cell recording) were pulled from borosilicate glass capillaries (Harvard) and filled with a solution containing: K^+^-gluconate 132 mM, EGTA/KOH 1 mM, MgCl_2_ 2 mM, NaCl 2 mM, Hepes 10 mM, MgATP 2 mM, and GTP 0.5 mM; pH 7.2 adjusted with TrisOH; osmotic pressure was adjusted to that of extracellular medium using saccharose. Under the extracellular medium condition used, the junction potential was calculated ∼ +16 mV using the JPCalc software in the PClamp suite (Axon Instruments) and corrections of membrane potentials were made a posteriori. When cell-attach recordings were needed, 15 MΩ electrodes were filled with filtered extracellular medium and the pipette potential maintained at 0 mV. The recorded current and voltage traces were digitized prior acquisition at 20 KHz using a Digidata 1320 (Axon Instruments). Off-line analysis was performed using MiniAnalysis (Synaptosoft) or pClamp-9 (Axon Instruments) softwares. When appropriate, changes in membrane resistance were determined from membrane current changes induced by a hyperpolarizing step of 10 mV under voltage-clamp.

### Determination of glutamate release using the Amplex red assay

Glutamate release was determined following a process inspired from Chapman and Zhou [46]. Granule cells cultured on 24-well plates were washed three times with 300 µl HBSS before being challenged with the indicated ET final concentrations. After 10 min incubation at 37°C, the medium was collected and stored appropriately for glutamate determination. Glutamate concentration was monitored three times for each sample using a cycling assay based on the ‘amplex red glutamic acid kit’ (Molecular Probes). Typically, reactions were conducted in 96-well plates. To each 50 µl sample was added 50 µl of reagent containing 0.25 U.ml^−1^ HRP, 0.08 U.ml^−1^ Glutamate oxydase, 0.5 U.ml^−1^ Glutamate pyruvate synthase, 0.2 mM L-alanine and 100 µM amplex red in 0.1 mM Tris buffer, pH 7.4. The cycling reaction was allowed to proceed at 37°C for 30 min and the increase in the resorufin fluorescence was measured using a fluorescence plate reader (Mithras LB 940, Berthold technologies) (530 nm excitation –590 nm emission). Determination of the total intracellular pool of glutamate was performed by lysing all the cells by a hypo-osmotic shock: the supernatant was removed and the cells were submitted to distilled water containing only 10 mM Tris, pH 7.4.

### Determination of lactate deshydrogenase release

The lactate des-hydrogenase released by ET treated cells was quantified using the Cytotoxicity Detection Kitplus (LDH) (Roche) on 96-well plates, following the manufacturer instructions. The assay was performed on the cell culture medium collected before the HBSS cell monolayer wash (basal release during the culture), on the same samples used for glutamate concentration quantification (LDH release due to toxin effect) and on the cell extract (total LDH) after hypo-osmotic shock. The reaction results are obtained by reading the optical density on a multi-plate reader Mithras LB 940 (Berthold, Germany).

### Other materials

Methyl-β-cyclodextrin was from Sigma-Aldrich. Tetrodotoxin (TTX, Latoxan), bicuculline methiodide, TEA, 4-AP (Sigma-Aldrich) were prepared as stock solutions in distilled water and CNQX (Sigma-Aldrich) was dissolved in DMSO prior dilution to the desired final concentration in the physiological solution immediately before the experiments.

### Statistical analysis

When adequate, results are presented as means ± SE from the indicated number *n* of separate experiments. Statistical comparisons were done using Khi^2^ or ANOVA tests as appropriate. When statistical differences were detected with ANOVA, multiple comparisons Bonferroni test was performed. A *p* value <0.05 was considered significant; *n.s.* denotes not significant comparison.
